# Shear Bond Strength of Silane‐Containing Universal Adhesives in Repairing Aged 3D‐Printed Provisional Restorations With Flowable Composite: An In Vitro Study

**DOI:** 10.1155/ijod/5245327

**Published:** 2025-12-22

**Authors:** Wisarut Prawatvatchara, Awutsadaporn Katheng, Paweena Kongkon, Santisuk Sombun, Piangkwan Saiprasert, Nawaporn Jittapiromsak

**Affiliations:** ^1^ Department of Prosthodontics, Faculty of Dentistry, Chulalongkorn University, Bangkok, Thailand, chula.ac.th; ^2^ Department of Restorative Dentistry, Faculty of Dentistry, Naresuan University, Phitsanulok, Thailand, nu.ac.th

**Keywords:** 3D-printed provisional restorative materials, repairability, shear bond strength, silane, universal adhesive

## Abstract

**Background:**

The repairability of provisional restorative materials affects the duration of the provisional treatment phase. This study evaluated the effects of various silane (Si)‐containing universal adhesives on the shear bond strength (SBS) of aged three‐dimensional (3D)‐printed provisional restorative materials.

**Methods:**

Seventy SBS specimens (20 mm diameter × 15 mm height) and 10 specimens for energy‐dispersive X‐ray (EDX) analysis (5 mm diameter × 3 mm height) were fabricated. All SBS specimens underwent thermocycling in artificial saliva at 5– 55 °C, with 60 s dwell time, using a thermocycler (SD Mechatronik, Germany). The SBS specimens were randomly divided into seven groups (*n* = 10 per group) on the basis of the repair method: C group (control), S group (Single Bond 2), SU group (Scotchbond Universal), SUP group (Scotchbond Universal Plus), CUQ group (CLEARFIL TRI‐S BOND Universal Quick), GPB group (G‐Premio Bond), and Si + S group (Silane Ultradent + Single Bond 2). The SBS test was performed using a universal testing machine (Instron, ElectroPulsTM E1000, England). Failure modes were analyzed for all debonding surfaces. Data was analyzed using one‐way analysis of variance (ANOVA) and Tukey’s HSD test (*p* < 0.05).

**Results:**

The highest SBS was observed in the Si + S group (21.06 ± 1.47 MPa), followed by SUP (19.43 ± 1.45 MPa), CUQ (16.14 ± 1.54 MPa), SU (15.71 ± 1.40 MPa), GPB (12.93 ± 1.56 MPa), S (12.59 ± 1.64 MPa), and C (8.20 ± 1.49 MPa). However, no significant difference in the SBS was observed between the Si + S and SUP groups. Additionally, there was no significant difference in SBS between S and GPB. Failure mode analysis revealed a correlation between the type of failure pattern and the SBS results.

**Conclusion:**

The SUP group, a silane‐containing universal adhesive, demonstrated high SBS comparable with the separate application of Si, suggesting it as a viable alternative in provisional material repair.

## 1. Introduction

Provisional or temporary restorations serve their purpose for a short to intermediate period prior to replacement with a permanent dental or maxillofacial rehabilitation prosthesis. They are intended to improve the esthetics of the restoration, and aid functional elements [1, 2]. In addition, they play a part in the maintenance of tooth position, ensuring the preservation of occlusion, protection of pulpal and periodontal health [3, 4], prevention of abutment migration, evaluation of the vertical dimension, and establishment of an appropriate occlusal scheme [1–4].

The provisional restorative materials must possess mechanical/physical traits and meet certain criteria, including biocompatibility [5, 6], nonirritation to dental pulp and tissues, low polymerization shrinkage [7], dimensional stability, internal fit [8], and high mechanical strength [9–11]. During the provisional phase of treatment, occlusion adjustment or defect repair of the provisional restoration is often performed. The capacity to adjust and repair provisional restorations is essential for accommodating changes in a patient’s condition or treatment planning, thus significantly improving the general effectiveness of prosthodontic treatments [3, 12, 13]. The frequent need for repair or relining of provisional restorations due to wear and breakage is a primary technical concern. Timely attention to these issues is essential to maintain the integrity of the restorations throughout the treatment process.

Computer‐aided design/computer‐aided manufacturing (CAD/CAM) technology has recently emerged as a novel method for creating provisional prostheses. CAD/CAM prostheses can be fabricated using additive manufacturing (AM) [14] or subtractive manufacturing (SM) [15]. AM methods enable the fabrication of three‐dimensional (3D) objects using a sequential layering method, allowing the accurate printing of complex geometries while reducing material waste and time consumption. There are seven categories of processes in AM, with vat photopolymerization being the most prevalent, including technologies such as stereolithography (SLA), digital light processing (DLP), and liquid crystal displays (LCD). In SM, a milling machine is used to shape prefabricated disks or blocks of materials into specific forms. The milling technique provides high‐quality dentures mainly because of highly cross‐linked polymerization [16]. Owing to their durability, precise fit, and improved esthetics, dentists and laboratories commonly use provisional restorations milled from polymethylmethacrylate (PMMA) blocks. However, challenges arise when discarded materials are reused or undercuts or inaccessible locations are addressed during machining. Recent findings indicate that AM methods, particularly 3D printing, offer greater accuracy in dental restoration fabrication than to subtractive methods [17].

Previous studies evaluated the mechanical properties of 3D‐printed provisional restorations, including the modulus of elasticity and wear resistance, which are comparable to those of conventional PMMA crowns [17]. Although 3D‐printed provisional crowns offer numerous advantages, they still require chairside customization, repairs, and adjustments. Several studies have investigated the influence of the type of repair material and surface treatment approach on the shear bond strength (SBS) of CAD/CAM provisional restorative materials and their repairability [18–21]. Chemical surface treatments have been thoroughly investigated for their effectiveness in enhancing the bonding of restorative materials [20, 21]. The application of silane coupling agent has been proven to be particularly effective in promoting durable bond strength. Silane interacts with silica‐based surfaces, forming a chemical bridge between the restoration and adhesive resin, enhancing the bond strength and clinical longevity [21].

Universal or multimode adhesives, which are used in only one component, either directly in a single solution, are classified as one‐step adhesive systems and employ self‐etching, etch‐and‐rinsing, or selective enamel etching based on the dentist’s requirements. Universal adhesives combine acidic primers and/or silane coupling agents with adhesive monomers [22–24].

Several studies have investigated the bonding strength of silane‐containing universal adhesives with aged resin composites [24, 25], hybrid ceramics [20, 26], and ceramic restoration materials [27]. However, studies on the effects of various silane‐containing universal adhesives on the reparability of aged 3D‐printed provisional restorative materials are lacking.

In addition, the precise elemental composition of 3D‐printed provisional restorative materials is often undisclosed by manufacturers. Since, the bonding effectiveness of silane is highly dependent on the presence and distribution of silica or other inorganic fillers, it is essential to characterize the elemental composition of the material. Therefore, energy‐dispersive X‐ray (EDX) analysis was performed to identify and map the elemental constituents of the 3D‐printed provisional restorative material. This information provides fundamental insights into surface chemistry that could influence the interaction with silane‐containing universal adhesives and explains the observed repair bond strengths.

This study aimed to investigate the effects of various silane‐containing universal adhesives on the SBS between aged 3D‐printed provisional restorative material and flowable resin composite. The null hypothesis was that there are no differences in SBS between each type of universal adhesive when flowable resin composite was bound to aged 3D‐printed provisional restorative material.

## 2. Materials and Methods

### 2.1. Experimental Design of the Investigation

The sample size was calculated to determine a suitable number of specimens using G^∗^Power Version 3.1.9.7. This study used the mean and sample size with 80% statistical power and a significance level of 0.05. The calculation indicated that 10 specimens should be collected for each group; hence, this study used 10 specimens for each of the seven SBS test groups and an additional 10 specimens for EDX analysis, resulting in a total of 80 specimens.

All the materials used in this investigation are listed in Table 1. The specimens were digitally designed using CAD software (3D Builder Version 18.0.1931.3; Microsoft Corporation, Washington, USA).

**Table 1:** Composition, manufacturers, and names of all the materials used in the investigation.

Materials	Brand name	Manufacturer (Lot no.)	Composition
Silane	Ultradent Silane	Ultradent Products Inc., South Jordan, UT, USA (B1221.2)	Isopropyl alcohol, *γ* methacryloxypropyltrimethoxysilane (*γ*MPTS)
3D‐printed provisional restorative materials	Asiga DentaTooth	ASIGA, Sydney, Australia (5000456)	7,7,9(or 7,9,9)‐trimethyl‐ 4,13‐dioxo‐3,14‐dioxa‐5,12‐ diazahexadecane‐1,16‐diyl bismethacrylate, tetrahydrofurfuryl methacrylate, diphenyl (2,4,6‐ trimethylbenzoyl) phosphine oxide
Adhesive	Single Bond 2 Adhesive	3M Adper (St. Paul, MN, USA) (10605601)	Ethyl alcohol, bisphenol A, bis‐GMA, silane treated silica, HEMA, glycerol 1,3 dimethacrylate, water, copolymer of acrylic and itaconic acids, UDMA, diphenyliodonium hexafluorophosphate, ethyl 4‐dimethyl aminobenzoate (EDMAB)
Universal adhesive	Scotchbond Universal Adhesive	3M ESPE (St. Paul, MN, USA) (30718A)	HEMA, bisphenol A, bis‐GMA, 2‐propenoic acid, 2‐methyl‐, reaction products with 1,10‐decanediol and phosphorous oxide, ethanol, water, silane treated silica, copolymer of acrylic and itaconic acid, camphorquinone, dimethylaminobenzoat(dimethylamino)ethyl methacrylate, 2,6‐di‐tert‐butyl‐p‐cresol, *γ* methacryloxypropyltrimethoxysilane (*γ*MPTS)
	Scotchbond Universal Plus Adhesive	3M ESPE (St. Paul, MN, USA) (9020276)	2‐Propenoic acid, 2‐methyl‐, diesters with4,6‐dibromo‐1,3‐benzenediol 2‐(2 hydroxyethoxy)ethyl 3‐hydroxypropyl diethers, 2‐hydroxyethyl methacrylate, 2‐propenoic acid, 2‐methyl‐, reaction products with 1,10‐decanediol and phosphorus oxide, 2‐propenoic acid, 2‐methyl‐, 3‐(triethoxysilyl)propyl ester, reaction products with silica and 3‐(triethoxysilyl)‐1‐propanamine, ethanol, water, synthetic amorphous silica, fumed, crystalline‐free, methacrylic Acid, 3‐(triethoxysilyl)propyl ester, camphorquinone, copolymer of acrylic and iItaconic acid, N, N‐dimethylbenzocaine, acetic acid, copper(2+) salt, monohydrate, optimized silane (*γ*MPTES; *γ*‐methacryloxypropyltriethoxysilane, APTES; 3‐(aminopropyl)triethoxysilane)
	CLEARFIL TRI‐S BOND Universal Quick	Kuraray, Okayama, Japan (740462)	10‐MDP, bis‐GMA, HEMA, hydrophilic amide monomer, colloidal silica, ethanol, silane coupling agent, dl‐Camphorquinone, accelerators, water, sodium fluoride
	G‐Premio Bond	GC Corp, Tokyo, Japan (2405070)	MDP, MDTP, 4‐MET, BHT, acetone, water, dimethacrylate monomer, photoinitiator, silica fillers
Flowable resin composite	Filtek Z350 XT	3M ESPE (St. Paul, MN, USA) (9571262)	Silane treated ceramic, bis‐GMA, bisphenol A polyethylene glycol diether dimethacrylate (BISEMA‐6), UDMA, silane treated silica, silane treated zirconia, PEGDMA, TEGDMA
Artificial saliva	Biotene Moisturizing Mouth	GlaxoSmithKline, Thailand (7288701)	Purified water, glyercin, xylitol, PEG‐60, hydrogenated castor oil, vinyl pyrrolidone with vinyl acetate copolymer, Flavor, sodium benzoate, xanthan gum, methylparaben, propylparaben, sodium saccharin, cetylpyridinium chloride

Abbreviations: BHT, butylated hydroxytoluene; Bis‐GMA, bisphenol A diglycidyl methacrylate; EDTA, ethylenediaminetetraacetic acid; HEMA, hydroxyethyl methacrylate; MDP, methacryloyloxydecyl dihydrogen phosphate; MDTP, methacryloyloxydecyl dihydrogen thiophosphate; PEGDMA, polyethylene glycol dimethacrylate; TEGDMA, triethylene glycol dimethacrylate; UDMA, urethane dimethacrylate; 4‐MET, 4‐methacryloyloxyethyl trimellitic acid.

### 2.2. Specimen Preparation for EDX Analysis

The EDX specimens were designed in a cylindrical shape with a diameter of 5 mm and a height of 3 mm and were produced by ASIGA (ASIGA composer, Sydney, Australia) using a DLP‐3D printer. The printing process involved a layer thickness of 50 *μ*m, resin color shade A2 (Asiga DentaTOOTH, ASIGA, Sydney, Australia), and a 45‐degree build orientation. Specimens were immersed in 98% isopropyl alcohol (KT Chemicals, Nishi, Osaka, Japan) for 60 s, then ultrasonically cleaned (Sonorex Super 10P/Banderlin, Austria) for 10 min to eliminate excess resin monomers, and then dried with compressed air. For postprocessing, the specimens were cured in a UV oven for 30 min (Otoflash G171; NK‐Optik GmbH, Germany). All specimens were polished with 400‐, 800‐, and 1000‐grit silicon carbide papers using a polishing machine. The specimens were cleaned in deionized water with an ultrasonic cleaner (Branson5210; Bransonic, CT, USA) for 10 min and stored in a dry place.

### 2.3. EDX Analysis

EDX was performed using a JSM‐IT200 (JEOL Ltd., Tokyo, Japan) electron microscope under a low vacuum of 80 Pa, landing voltage of 15.0 kV, and magnification of ×500. An EDX analysis was conducted to evaluate the elemental compositions of the specimens. EDX was used to identify, quantify, and map the elemental content. This analysis offers insights into the surface and elemental compositions that influence the bonding of 3D‐printed provisional restorative materials.

### 2.4. Specimen Preparation for SBS Testing

**Figure 1:**
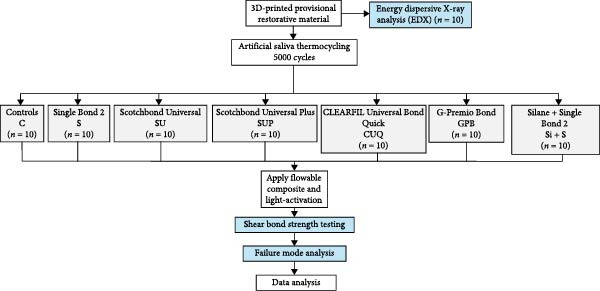
The diagram shows the experimental design.

The SBS specimen files were cylindrical with a diameter of 20 mm and a height of 15 mm, modified from the International Organization for Standardization (ISO 10477:2020) standards for dental polymer‐based crown and veneering materials [28]. The experimental design is illustrated in Figure 1.

Seventy specimens were produced using ASIGA, as described for the previous EDX specimens. The specimens were measured using a digital Vernier caliper (Mitutoyo CD15; Mitutoyo Co., Kawasaki, Japan) for accurate dimensional assessment. A polishing machine was used to polish all the specimens with silicon carbide paper with grit sizes of 400, 800, and 1000. Specimens exhibiting defects, fractures, or voids were discarded after inspection using a 40× stereomicroscope (Olympus SZ61, Olympus Optical Co., Tokyo, Japan). The specimens were finally cleaned in distilled water using an ultrasonic cleaner for 10 min and thereafter submerged in deionized water at 37 °C for 24 h in an incubator.

### 2.5. Thermocycling of Artificial Saliva in the Aging Process

All the SBS specimens underwent thermocycling to simulate intraoral aging conditions. The thermocycling protocol involved 5000 cycles of alternating temperature exposure, ranging from 5°C to 55°C, using a thermocycler (SD Mechatronik, Feldkirchen‐Westerham, Germany). Each cycle included a dwell time of 60 s in artificial saliva (Biotène Moisturizing Mouth Rinse; GlaxoSmithKline, Thailand) to mimic the oral environment. After the aging procedure, all specimens were meticulously cleaned with deionized water to eliminate residual saliva, air‐dried to remove surface moisture, and placed in a desiccator to prevent further environmental effects before testing.

### 2.6. Bonding Procedures

After the aging procedure was completed, the SBS specimens were randomly divided into seven categories according to the repair method. To control the bonded surface area, a 2‐mm hole on polyethylene tape (thickness of 0.1 mm) was placed at the center of the specimen surfaces prior to the application of the adhesive agent. Each group was subsequently categorized into seven groups (*n* = 10 in each group) based on the adhesive application technique employed, according to the manufacturer’s instructions.

1. In group C (control), no surface treatment was applied, and the specimens received no adhesive application.
2. In the S group (Single Bond 2), the adhesive was applied to each specimen using a microbrush for 20 s and air‐dried for 5 s with a triple syringe, then light‐activated using an LED device (Elipar S 10, 3M ESPE, St. Paul, MN, USA) for 10 s.
3. In the SU group (Scotchbond Universal), the adhesive was applied to each specimen using a microbrush for 20 s and air‐dried for 5 s with a triple syringe, and light‐activated using an LED device for 10 s.
4. In the SUP group (Scotchbond Universal Plus), the adhesive was applied to each specimen using a microbrush for 20 s, air‐dried for 5 s with a triple syringe, and light‐activated using an LED device for 10 s.
5. In the CUQ group, CUQ (CLEARFIL TRI‐S BOND Universal Quick) was applied to each specimen using a microbrush, air‐dried for 5 s using a triple syringe, and light‐activated using an LED device for 5 s.
6. In the GPM group (G‐Premio Bond), the adhesive was applied to each specimen using a microbrush for 10 s, air‐dried for 5 s using a triple syringe, and light‐activated using an LED device for 5 s.
7. In the Si + S group (Silane Ultradent + Single Bond 2), the specimens were gently applied to the silane agent (Silane Ultradent) with a microbrush in a thin layer and allowed to vaporize completely for 10 s before air drying with a triple syringe. Subsequently, Single Bond 2 adhesive was applied in the same manner as that in the S group.

**Figure 2:**
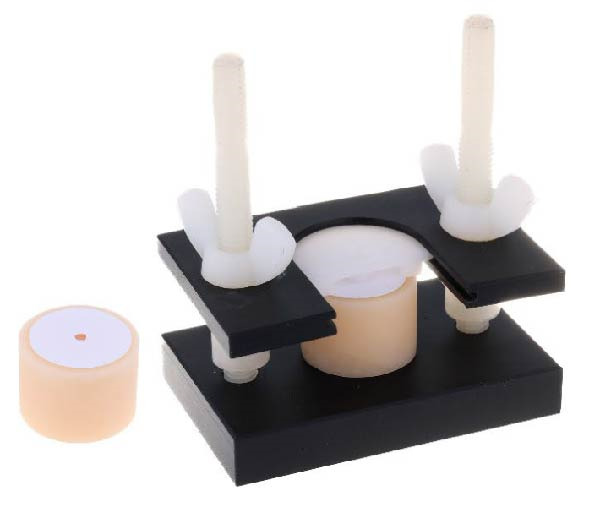
The specimens are secured in an Ultradent jig and detachment mold to ensure a uniform bonding surface.

After bonding each specimen group, the ultradent polyethylene jig and detached mold (Ultradent Products, South Jordan, UT, USA), which had an internal diameter of 2.38 mm and a height of 2 mm, were fixed to the bonded surface, as shown in Figure 2. A flowable resin composite (Filtek Z350 XT; 3M ESPE, St. Paul, MN, USA) was injected into the cavity of a detachable piece of the mold and polymerized according to the manufacturer’s instructions. After completion of the adhesive and resin composite buildup procedures, all specimens were stored in deionized water at 37 °C for 24 h to allow postcure stabilization prior to SBS testing.

### 2.7. Notched‐Edge SBS Testing

The SBS was evaluated by conducting tests on the repaired specimens using a universal testing machine (Instron, ElectroPulsTM E1000, Leicester, UK). The specimens were affixed to a jig in a universal testing machine and exposed to shear force using a notch‐edge shear blade at a crosshead speed of 0.5 mm/min, along a direction parallel to the bonded interface until failure occurred.

### 2.8. Mode of Failure Analysis

Following SBS testing, the debonded surfaces of all specimens were inspected for failure using a 40× magnification stereomicroscopy (Olympus SZX16, Olympus, Tokyo, Japan).

Fracture modes were classified according to established criteria as follows: adhesive fracture when failure occurred exclusively at the bonding interface (i.e., no residual 3D‐printed provisional resin or resin composite remained on either debonded surface); cohesive fracture when failure occurred within a single substrate (either the 3D‐printed provisional resin or the resin composite) with the interface remaining intact; and mixed fracture when both interfacial and substrate failures were present within the same specimen [29–31].

### 2.9. Statistical Analysis

Statistical analyses were performed using IBM SPSS software version 22 (SPSS Inc., Chicago, IL, USA). The Shapiro–Wilk test was used to assess the assumption of normality, and Levene’s test was used to evaluate variance homogeneity. One‐way analysis of variance (ANOVA) was performed, followed by Tukey’s post‐hoc comparison test to evaluate differences between groups. The results were considered statistically significant at *p* < 0.05.

## 3. Results

### 3.1. EDX Analyses

**Figure 3:**
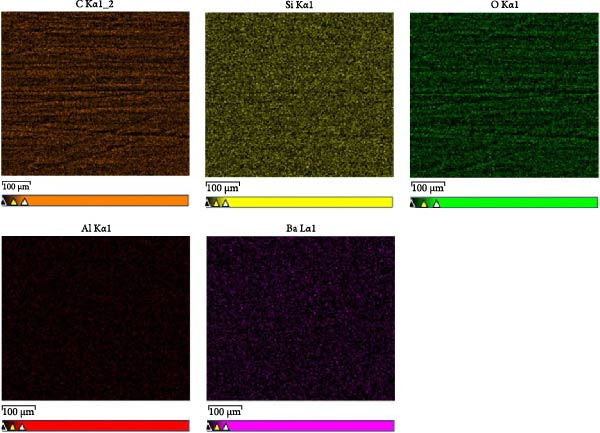
EDX mapping analysis of the 3D‐printed provisional restorative material (C, O, Si, and Al).

The EDX analysis of the 3D‐printed provisional restorative material revealed the following elemental compositions: carbon (C) 58.89 ± 2.02 wt%, oxygen (O) 33.25 ± 1.24 wt%, silicon (Si) 4.04 ± 0.27 wt%, and aluminum (Al) 0.37 ± 0.03 wt%. The elemental compositions are listed in Table 2. Elemental mapping revealed consistent and extensive elemental distribution in the 3D‐printed provisional restorative material (Figure 3).

**Table 2:** EDX results of 3D‐printed provisional restorative materials with relative values expressed as weight percentages.

Element	Weight (%)
Carbon (C)	58.89 ± 2.02
Oxygen (O)	33.25 ± 1.24
Silicon (Si)	4.04 ± 0.27
Barium (Ba)	3.03 ± 0.12
Rubidium (Rb)	0.48 ± 0.02
Aluminum (Al)	0.37 ± 0.03
Calcium (Ca)	0.14 ± 0.01

### 3.2. SBS

The SBS values of the experimental groups indicated differences in bonding efficacy, as shown in Table 3. Statistical analysis revealed that although the Si + S group showed the highest SBS, the difference compared with the SUP group was not statistically significant. Similarly, the SU and CUQ groups showed no significant difference. No significant difference was also observed between the GPB and S groups. The C group exhibited the lowest SBS among all tested groups.

**Table 3:** Mean and standard deviation variation in shear bond strength values (MPa) for each group.

Group	Means (SD) of SBS
C	8.20 (1.49)^d^
S	12.59 (1.64)^c^
SU	15.71 (1.40)^b^
SUP	19.43 (1.45)^a^
CUQ	16.14 (1.54)^b^
GPB	12.93 (1.56)^c^
Si + S	21.06 (1.47)^a^

*Note:* The different superscripted lowercase letters indicate significant differences across groups, whereas *p* < 0.05.

### 3.3. Mode of Failure Analysis

**Figure 4:**
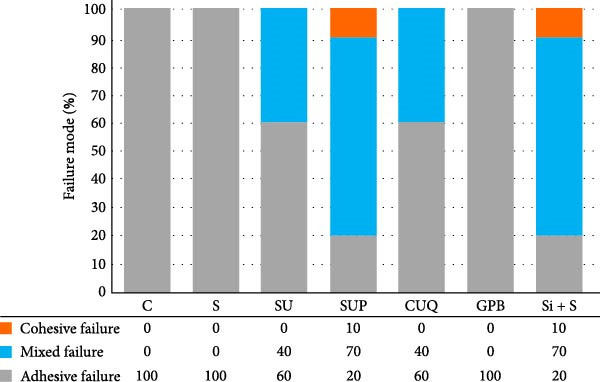
The percentage distributions of failure modes among groups.

The failure modes observed during testing are illustrated in Figure 4. Cohesive failure occurred only in the SUP and Si + S groups (10%). All cohesive failures occurred within the 3D‐printed provisional restorative material. Mixed failures were observed in the SU (40%), SUP (70%), CUQ (40%), and Si + S groups (70%). Adhesive failure occurred exclusively in the C, S, and GPB groups (100%), followed by the SU (60%), whereas the SUP and Si + S groups had the lowest incidence (20%). The failure modes of the specimens are shown in Figure 5.

Figure 5Failure mode assessed in this research; the dashed line represents the fracture boundary: (a) adhesive failure; (b) mixed failure; (c) cohesive failure; P: provisional restorative material; i: bonding interphase.

**Figure:**
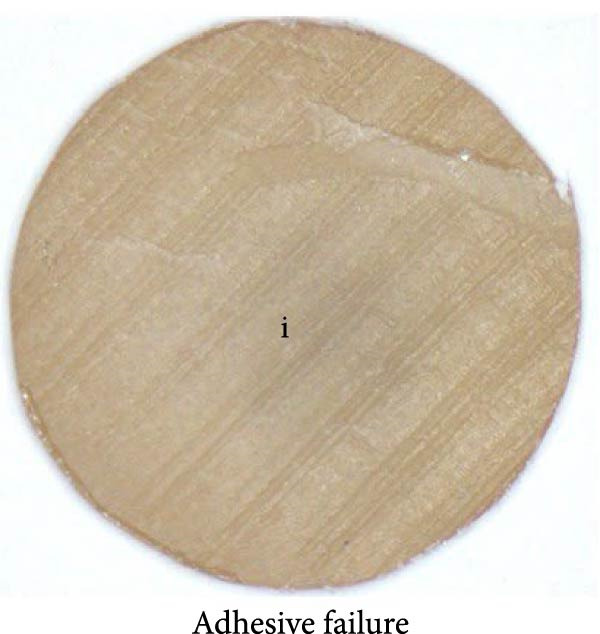
(a)

**Figure:**
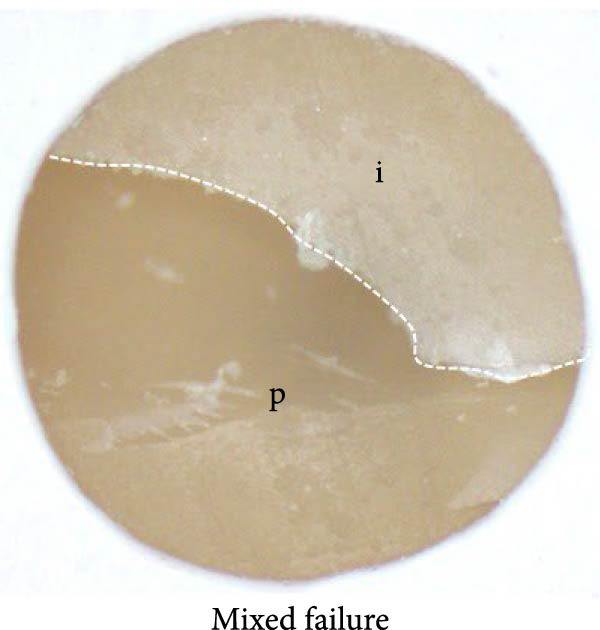
(b)

**Figure:**
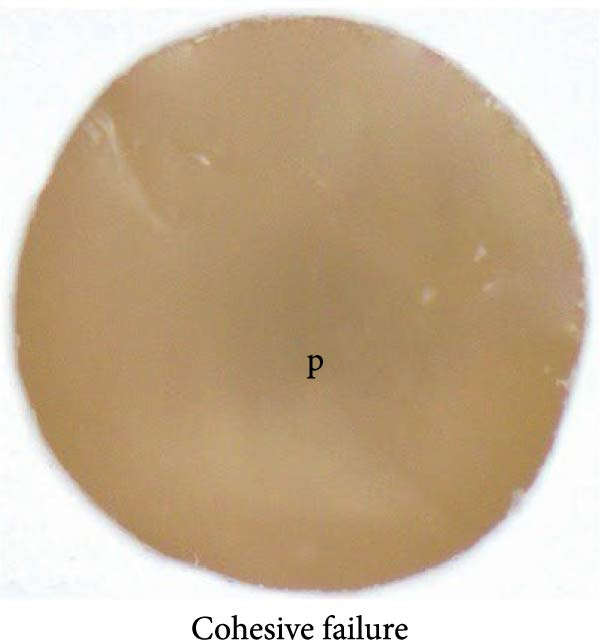
(c)

## 4. Discussion

This study aimed to assess the repairability, particularly the SBS, of aged 3D‐printed provisional restorative materials to flowable resin composites and to analyze the surface characteristics of these materials. These findings indicate variations in SBS among different universal adhesives, leading to the rejection of the null hypothesis of this investigation. This study also investigated the effect of different silane types and silane‐free universal adhesives on aged 3D‐printed provisional restorative materials.

This investigation attempted to determine the elemental composition by EDX studies, identifying the type and amount of elements indicative of the inorganic fillers, owing to the unavailability of manufacturer information. EDX analysis revealed the presence of a silica‐based filler. Furthermore, elemental mapping revealed a consistent and extensive distribution of elements in these materials, which positively affected chemical bonding via silane [32]. The presence of silica‑containing fillers in the 3D‑printed provisional resin indicating that hydroxylated glass/silica surfaces are available for silane coupling. Upon hydrolysis, trialkoxysilanes form silanols that condense with surface ‐Si‐OH to create a siloxane network (Si‐O‐Si) on the filler, while the methacrylate end of the silane copolymerizes with the adhesive/composite matrix during light activation. This dual reactivity establishes a molecular bridge across the filler–resin interface and provides a chemical rationale for the higher SBS and the shift from purely adhesive to mixed/cohesive failures in silane‑containing protocols observed in this study. Beyond filler coupling, the resin matrix of the printed material is a dimethacrylate network. The solvents in universal adhesives (ethanol/water or acetone) transiently plasticize the top layer, allowing monomer diffusion. Consequently, hydrolyzed silane and low molecular weight methacrylates can interpenetrate and copolymerize with the aged network, forming a covalently integrated interphase.

Clinical representativeness of aging. Although in vitro thermocycling cannot reproduce all intraoral variables, the present regimen (5 °C/55 °C; 60 s dwell; 5000 cycles) targets approximately 6 months of service according to the commonly cited research [33]. The use of artificial saliva aims to combine hydrothermal and electrolyte effects rather than water alone, which is pertinent for polymer networks and filler/resin interfaces [34, 35]. Importantly, this timescale represents the long‐term clinical service expected provisional, where repair decisions are made within months rather than years [1–4]. Hence, the present aging condition is appropriate for the intended clinical window while acknowledging that mechanical fatigue and pH/enzymatic challenges in vivo are not fully replicated.

Artificial aging is a laboratory procedure used to simulate oral conditions and assess changes in material quality. Numerous studies have immersed materials in liquids, such as water, red wine [36], or artificial saliva [34, 37], or exposed them to thermocycling [32–35, 37]. However, artificial saliva thermocycling, which integrates hydrothermal and chemical electrolyte effects, has not been widely used. Dentin and enamel [37], as well as polyurethane dental aligners [35], have been subjected to thermocycling in artificial saliva to evaluate the effects of aging. According to the SBS results, the Si + S group had the highest SBS, exceeding those of the other universal adhesive groups. This result is attributed to the separation of the silane application from the adhesive, which enhances its efficacy. In general, dental silane comprises organofunctional trialkoxysilanes, such as *γ*‐methacryloxypropyltrimethoxysilane (*γ*MPTS) and *γ*‐methacryloxypropyltriethoxysilane (*γ*MPTES), which are diluted and solubilized in ethanol and water at specific pH values to facilitate the hydrolysis of silane [32]. However, previous studies have shown that silanes are relatively stable compared with organotrialkoxysilanes when the pH is between 4 and 5 [38]. Many universal adhesives have been formulated by including silane, primarily *γ*MPTS, to facilitate the bonding of inorganic materials, including ceramic restorations and inorganic fillers. In addition to silane, several universal adhesives use 10‐methacryloxydecyl phosphate (MDP) as an acidic functional monomer. However, previous studies have shown that the pH of commonly used MDP varies between 2.0 and 2.7 [38, 39]. Consequently, the pH of silane‐containing universal adhesives, including MDP, is often low, as exemplified by SBU (pH 2.7) and CUQ (pH 2.3). Several studies have indicated that the acidity of MDP in silane‐containing universal adhesives may negatively affect the efficiency of *γ*MPTS [38–41]. Consequently, given the acidic environment, the efficacy and long‐term stability of the silane included in the universal adhesive are important concerns.

In our study, the silane‐containing universal adhesive containing *γ*MPTS, which was present in the SU and CUQ groups, showed a lower SBS than the Si + S group. These findings could indicate the decreased effectiveness of *γ*MPTS in acidic environments.

For silica‐based glass ceramics, several studies show that relying on the silane premixed in a universal adhesive yields lower bond strengths than applying a separate silane primer step on the ceramic surface, because the acidic, water‐containing environment and acidic phosphate monomers (e.g., MDP) in universal adhesives can destabilize *γ*MPTS and impede the formation of stable interfacial Si‐O‐Si bonds [38, 40–43]. This has been attributed to the self‐condensation of functional silanols owing to the molecular instability of *γ*MPTS in aqueous acidic solutions, such as universal adhesives. The self‐condensation of silanol leads to the formation of a thick silane oligomer, resulting in bond failure inside the silane layer, which significantly decreases bond strength [38, 39, 41]. However, when the *γ*MPTS silane‐containing universal adhesives (SU and CUQ groups) were compared with the silane‐free universal adhesive (GPB group), the SBS values of the SU and CUQ groups were significantly higher than those of the GPB group. Moreover, the GPB group, categorized as a silane‐free universal adhesive, demonstrated no significant difference compared with the S group, which was considered a silane‐free adhesive. Although GPB group contain functional components, their chemistries are not optimized for the silica‑bearing, 3D‑printed provisional restorative material, favoring silane coupling rather than phosphate/acid‑monomer interactions. GPB relies on MDP/MDTP/4‑MET, which preferentially interact with hydroxyapatite or metal‑oxide substrates [44, 45], while S is a HEMA‑rich etch‑and‑rinse adhesive without a dedicated silane step. consistent with the total adhesive failures and low SBS observed for S and GPB.

The SBS of the SUP group was higher than that of the other universal adhesive groups. Although the SUP group had a lower SBS value than the Si + S group, the difference was not significant. The SUP adhesive comprises an optimized silane, which includes two types of silane: organofunctional trialkoxysilane (such as *γ*MPTES) and 3‐(aminopropyl)triethoxysilane (APTES). Similar to *γ*MPTS, *γ*MPTES has a methacrylate group that facilitates interactions with the restorative resin composite. Nevertheless, the ‐Si‐O‐C_2_H_5_ group in *γ*MPTES underwent hydrolysis at a slower rate than the ‐Si‐O‐CH_3_ group in *γ*MPTS. This effect may decrease the self‐condensation of silanol groups [42, 43]. Aminosilane compounds, such as APTES, are bifunctional silanes that are extensively used in biotechnology and contain amino‐terminal groups and reactive alkoxy moieties [43, 46, 47], allowing the silanol groups to react with ceramic surfaces or silica substrates in composite materials. Additionally, hydrogen bonds between two adjacent amino groups may form and help stabilize the hydrolyzed silanols on the surface of silica‐based substrates [43, 47].

In the failure mode analysis, cohesive failure was only observed in the Si + S and SUP groups. This finding indicates the efficiency of the optimized silane‐containing universal adhesive, which can improve the bonding between the resin composite and aged 3D‐printed provisional restorative materials, and is correlated with the SBS results. Cohesive failure occurred only in the 3D‐printed provisional restorative materials, indicating a weakness or defect in the layered structure of the UV‐polymerized resin materials. Mixed failure was also observed in silane‐containing universal adhesives, such as the SU, SUP, CUQ, and Si + S groups. Complete adhesive failure was entirely visible in the C, S, and GPB groups, indicating repair in the absence of silane.

Although no study has evaluated the effect of optimized silane‐containing universal adhesives on the repair of aged 3D‐printed provisional restorative materials, the findings of this investigation are similar to those of prior studies on aged resin composites [25, 26, 42], hybrid ceramics [27], and ceramics [28, 41–43]. The results of this investigation suggest that the use of an optimized silane‐containing adhesive to repair 3D‐printed provisional restorative materials can replace separate silane applications. This is because the optimized adhesive maintained a high bond strength while simplifying the procedure by reducing the number of adhesive steps and ultimately decreasing the chairside time.

In clinical scenarios, there are no established recommendations for repair bond strengths of provisional restorations. Previous studies indicated that the bond strength for repairs between aged composites and new composites should reach 18 MPa to be considered clinically acceptable for maintaining occlusal function [48, 49]. In this experiment, only the Si + S and SUP groups exhibited repair bond strengths above the previously suggested values.

A limitation of the present investigation is that it only evaluated the bond strength related to the chemical bonding from silane in different universal adhesives. In addition, all specimens were sequentially polished with silicon carbide papers of 400, 800, and 1000 grit to standardize the surface roughness and eliminate irregularities from the printing process, ensuring comparability among groups. However, such a polishing protocol does not directly replicate clinical repair procedures of provisional restorations, where surface roughening methods such as rotary instruments or sandblasting are more common. Mechanical surface treatment methods, such as airborne‐particle abrasion or sandblasting, which increase surface roughness, were excluded from this study. However, in clinical practice, integrating synergistic combinations of mechanical and chemical surface treatments that ensure a stable and long‐lasting bond at the molecular level may achieve more predictable bonding outcomes, ultimately enhancing repair. Furthermore, this study employed only one 3D‐printed provisional restorative material. The elemental composition of each brand varies which could lead to different repair outcomes. Finally, the repairability of the bonding adhesive was not entirely determined by SBS alone. Several clinical parameters are required to predict clinical repair performance, including flexural strength, fatigue resistance under thermomechanical cycling, and color stability after clinical use; together these parameters complement bond‑strength data and better predict in vivo durability.

Further investigations should evaluate synergistic combinations of mechanical and chemical surface treatments. More brands and various types of 3D‐printed resin composites should be integrated into evaluations, and assessments for predicting repairability performance, such as the bond performance of the postaging repair procedure, should be enhanced.

These areas of investigation will improve our understanding of the performance of various silanes in universal adhesives and the repairability of aged 3D‐printed provisional restorative materials. Subsequent prospective studies should assess alternative chemical surface treatments before the use of universal adhesives. Furthermore, the influence of chemical cleaning agents on saliva contamination should be analyzed in subsequent studies, providing greater representation and comprehension in clinical scenarios.

## 5. Conclusion

In evaluating the reparability of aged 3D‐printed provisional restorative material based on the SBS results, the optimized silane‐containing universal adhesive demonstrated superior SBS compared to other universal adhesives and similar applications of separating silanes. In clinical situation, the application of an optimized silane‐containing universal adhesive can minimize the number of clinical procedural steps and reduce treatment duration, while maintaining SBS comparable to that of silane application in the repair of 3D‐printed provisional restorative materials.

## Ethics Statement

The authors have nothing to report.

## Consent

The authors have nothing to report.

## Disclosure

A preprint has previously been published. All the authors reviewed and endorsed the text and confirmed its accuracy.

## Conflicts of Interest

The authors declare no conflicts of interest.

## Author Contributions

Wisarut Prawatvatchara: developed the foundational research design and contributed to the manuscript. Awutsadaporn Katheng: developed the foundational research design, performed data analysis, and quality evaluation. Paweena Kongkon and Piangkwan Saiprasert: conducted sample preparation and characterization. Wisarut Prawatvatchara, Awutsadaporn Katheng, Paweena Kongkon, Santisuk Sombun, Piangkwan Saiprasert, and Nawaporn Jittapiromsak: manuscript formatting and revision. Paweena Kongkon and Nawaporn Jittapiromsak: conducted a review of the paper.

## Funding

The authors received no specific funding for this work.

## Data Availability

The data that support the findings of this study are available upon request from the corresponding author. The data are not publicly available due to privacy or ethical restrictions.
